# LINC00221 suppresses the malignancy of children acute lymphoblastic leukemia

**DOI:** 10.1042/BSR20194070

**Published:** 2020-05-04

**Authors:** Man Huang, Jiajia Zheng, Yongya Ren, Jingjing Zhu, Linbing Kou, Jinhong Nie

**Affiliations:** 1 Department of Hematology, Suzhou Dushuhu Public Hospital (Dushuhu Public Hospital Affiliated to Soochow University, The First Affiliated Hospital of Soochow University, Dushuhu Branch), Suzhou, Jiangsu 215000, China; 2 Department of Hematology/Oncology, Children’s Hospital of Soochow University, Suzhou, Jiangsu 215000, China; 3 Department of Hematology, Soochow Hopes Hematonosis Hospital, Suzhou, Jiangsu 215000, China

**Keywords:** acute lymphoblastic leukemia, ATP2A2, LINC00221, miR-152-3p

## Abstract

As the most common malignant disease in childhood, children acute lymphoblastic leukemia (ALL) is a heterogeneous disease caused by the accumulated genetic alterations. Long non-coding RNAs (lncRNAs) are reported as critical regulators in diseases. GEPIA database indicated that long intergenic non-protein coding RNA 221 (LINC00221) was conspicuously down-regulated in acute myeloid leukemia. However, its expression pattern in ALL has not been revealed. This work was carried out to study the role of LINC00221 in ALL cells. Quantitative real-time PCR (qRT-PCR) quantified LINC00221 expression in ALL cells. The function of LINC00221 in ALL was determined by ki-67 immunofluorescence staining, EdU, TUNEL, JC-1, and caspase-3/8/9 activity assays. RNA pull down and Ago2-RNA immunoprecipitation (RIP) assays investigated the interaction between miR-152-3p and LINC00221 or ATPase sarcoplasmic/endoplasmic reticulum Ca2^+^ transporting 2 (ATP2A2). Our study revealed the low expression of LINC00221 in ALL cells. Subsequently, LINC00221 was verified to bind with miR-152-3p. Moreover, functional assays pointed out that LINC00221 overexpression posed anti-proliferation and pro-apoptosis effects in ALL cells, and these effects could be separately reversed by miR-152-3p up-regulation. Afterward, LINC00221 was revealed to regulate ATP2A2 expression via sponging miR-152-3p. Additionally, ATP2A2 was verified to involve in regulating LINC00221-mediated ALL cell proliferation and apoptosis. In conclusion, LINC00221 suppressed ALL cell proliferation and boosted ALL cell apoptosis via sponging miR-152-3p to up-regulate ATP2A2.

## Introduction

Acute lymphoblastic leukemia (ALL), a heterogeneous disease occurring especially in children, is featured by abnormal accumulation of T or B lymphoblasts in bone marrow. Children ALL is one of the commonest diseases with high incidence in children between 2 and 5 years old, and takes up approximately 25% of all childhood diseases [1,2]. Although remarkable improvements have been made in ALL treatment, non-response to drugs is still the major cause of death in ALL patients [3]. Thus, it is quite urgent to further research the potential mechanism underlying ALL for improving ALL treatment.

Long non-coding RNAs (lncRNAs) are a group of non-coding RNAs with a length more than 200 nucleotides. Recently, lncRNAs are emerging as critical mediators in various cancers, including ALL. For example, lncRNA PVT1 facilitates ALL development [4]. LncRNA NALT, a neighbor of NOTCH1, modulates NOTCH1 pathway and promotes ALL cell proliferation [5]. LncRNAs of NONHSAT027612.2 and NONHSAT134556.2 play important roles in ALL pathogenesis via regulation on immune response-associated pathways [6]. LncRNAs are widely reported to involve in ceRNA pattern that lncRNAs sponge miRNAs to modulate expression of miRNA targets. The ceRNA pattern in cancer is commonly reported. For instance, lncRNA PSMG3–AS1 serves as a sponge of miR-143-3p to regulate COL1A1 expression, thus enhancing proliferation and migration ability of breast cancer cells [7]. COL1A1-014 functions as a ceRNA in gastric cancer development via up-regulating CXCL12 and CXCR4 through interacting with miR-1273h-5p [8]. Up-regulation of lncRNA HOTAIR promotes PRAF2 expression via acting as a sponge of miR-326 in cutaneous squamous cell carcinoma [9]. LncRNA NEAT1 contributes to lung cancer cell proliferation and invasion via NEAT1/miR-1224/KLF3 ceRNA pattern [10].

Long intergenic non–coding RNA 00221 (LINC00221) was reported to enhance the cisplatin resistance of non-small cell lung cancer [11]. Also, LINC00221 is a prognostic biomarker for patients with hepatocellular carcinoma [12]. Besides, GEPIA database (http://gepia.cancer-pku.cn/) indicated that LINC00221 was conspicuously down-regulated in acute myeloid leukemia. However, the expression pattern and functional role of LINC00221 in ALL are still uncovered. Thus, the study was designed to explore the function and underlying mechanism of LINC00221 in ALL cells.

## Materials and methods

### Cell culture

Human ALL cell lines (Jurkat, CCRF-CEM, CEM/C1, 1301) and normal HEK 293T cell line were available from the ATCC (Manassas, VA) and cultivated in 5% CO_2_ at 37°C. Cell samples were all maintained in RPMI-1640 medium, with 10% FBS (HyClone, Logan, UT) and 1% penicillin–streptomycin as supplements.

### RNA isolation and quantitative real-time PCR

Total RNAs were acquired from cultured cell samples employing TRIzol reagent (Invitrogen Life Technologies Co, CA) for cDNA synthesis. Quantitative real-time PCR (qRT-PCR) was implemented with StepOne™ Real-Time PCR System (Applied Biosystems, Carlsbad, CA) as per the direction on the reagents. GAPDH or U6 served as the internal reference, with the 2^−ΔΔ*C*_t_^method used to calculate the relative gene expression.

### Plasmid transfection

Plasmid transfection was performed in six-well plates for 48 h in CCRF-CEM and CEM/C1 cell samples with Lipofectamine 2000 (Invitrogen). A total of 75 pmol of miR-152-3p-mimics and NC mimics, 75 pmol of miR-152-3p-inhibitor and NC inhibitor, pcDNA3.1-LINC00221 and pcDNA3.1-NC, the shRNAs specific to LINC00221 or ATPase sarcoplasmic/endoplasmic reticulum Ca2^+^ transporting 2 (ATP2A2) and NC-shRNAs, all these transfection plasmids were available from GenePharma Company (Shanghai, China). Bio-triplicates were conducted.

### RNA pull down

RNA pull down assay was implemented in CCRF-CEM and CEM/C1 cells by the utilization of Pierce Magnetic RNA-Protein Pull-Down Kit (Thermo Fisher Scientific, Waltham, MA). The protein extracts from cultured CCRF-CEM and CEM/C1 cells were prepared for incubation with the biotinylated RNA probes or non-biotinylated RNA probes as negative control (NC). The streptavidin agarose magnetic beads were added, and RNA enrichments were monitored by qRT-PCR after 1 h. Bio-triplicates were conducted.

### Luciferase reporter assay

Wild-type and mutated reporter vectors of LINC00221-WT/MUT or ATP2A2-WT/MUT, which covered the wild-type or mutated miR-152-3p binding sites were synthesized using pmirGLO Vector (Promega, Madison, WI). CCRF-CEM and CEM/C1 cells were co-transfected individually with luciferase vectors and indicated transfection plasmids. The luminescence changes were determined in each group using Dual-Luciferase Reporter Assay System (Promega) after 48 h. Bio-triplicates were conducted.

### RNA immunoprecipitation

Cell extracts from RNA immunoprecipitation (RIP) lysis buffer were reaped and incubated in the RIP buffer adding the magnetic beads-coated with human Ago2 antibody or control IgG antibody (NC; Millipore, Billerica, MA) for 4 h. qRT-PCR was conducted after adding magnetic beads. Bio-triplicates were conducted.

### Cytoplasm/nucleus fraction

Based on the user manual, the PARIS Kit (Invitrogen) was applied to acquire the cell cytoplasm/nucleus fraction. For conducting qRT-PCR, GAPDH and U6 served as the fractionation indicators of cell cytoplasm and cell nucleus. Bio-triplicates were conducted.

### FISH

The permeabilized cell samples were prepared for incubating with the specific LINC00221 FISH probe (Ribobio, Guangzhou, China) all night in hybridization solution. Samples were then counterstained with DAPI kit and analyzed with Olympus fluorescence microscope (Tokyo, Japan). Bio-triplicates were conducted.

### Immunofluorescence staining

ALL cell lines were fixed with 4% paraformaldehyde and blocked in 5% BSA for immunofluorescence (IF) assay. The primary antibody against ki-67 and the secondary antibody were available from Abcam (Cambridge, MA). After washing, samples in the slides were cultured in DAPI solution for 10 min and analyzed via Olympus fluorescence microscope. Bio-triplicates were conducted.

### EdU staining

Proliferation ability of ALL cells was detected by EdU staining kit (Ribobio) after transfection in 96-well plates. A total of 100 μl of EdU medium (50 μM) was added for 3 h, followed by fixing and permeabilizing. Proliferative cells were observed through Olympus fluorescence microscope after DAPI staining. Bio-triplicates were conducted.

### TUNEL staining

Forty-eight hours post-transfection, fixed ALL cells were collected and permeated with 0.1% Triton X-100. After washing in PBS, TUNEL reaction mixture was used for 1 h as per the instructions of the TUNEL staining kit. Olympus fluorescence microscope was applied for observing. Bio-triplicates were conducted.

### JC-1 assay

Samples of ALL cells were centrifuged at room temperature for 5 min, and mixed with 10 mm of JC-1 dye for 30 min. The cultured mixture was washed for imaging under fluorescence microscope. Bio-triplicates were conducted.

### Caspase activity detection

The capase-3/8/9 activity kits were available from Solarbio (Beijing, China) to determine the capase-3/8/9 activities in ALL cell lysates in light of the user manual. The protein extracts were placed in 96-well plates adding the caspase substrate and reaction buffer for 4 h. The mixture was processed with microplate reader at wavelength of 405 nm. Bio-triplicates were conducted.

### Statistical analyses

Bio-triple replications were required for each assay in the present study. The measurement data were shown as the mean ± standard deviation (SD) and analyzed through Prism 6.0 software. Comparison between two groups was processed with Student’s *t* test, with one-way analysis of variance among multiple groups. *P*-value <0.05 was seen significant statistically.

## Results

### LINC00221 was down-regulated in ALL cells and bound to miR-152-3p

**Figure 1:**
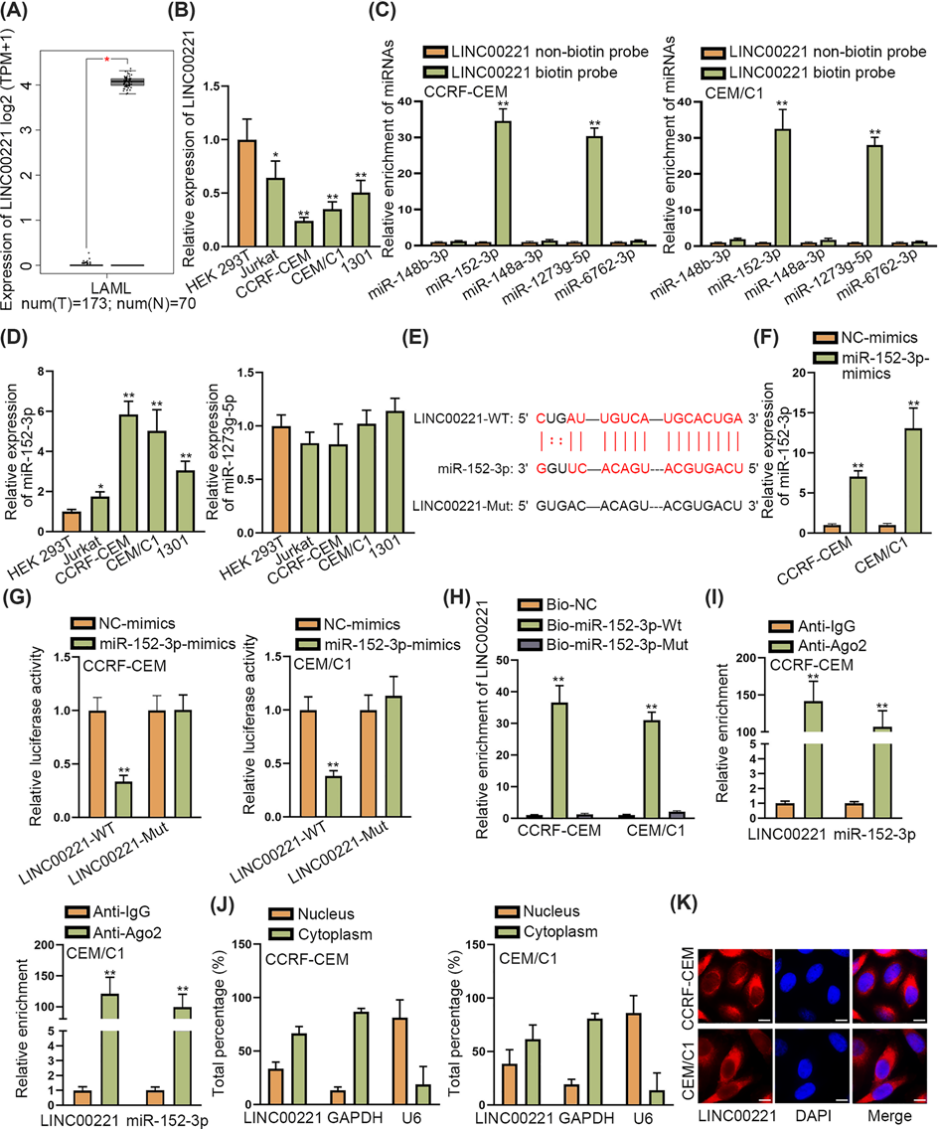
LINC00221 was down-regulated in ALL cells and bound miR-152-3p (**A**) GEPIA database demonstrated down-regulation of LINC00221 in AML tissues. (**B**) qRT-PCR detected expression level of LINC00221 in ALL cells and HEK-293T cells (control). (**C**) RNA pull down assay examined enrichment of candidate miRNAs pulled down by biotin-labeled LINC00221. (**D**) qRT-PCR detected expression level of miR-152-3p and miR-1273g-5p in ALL cells and HEK-293T cells. (**E**) Binding sites of wild/mutant LINC00221 and miR-152-3p. (**F**) qRT-PCR examined miR-152-3p expression in ALL cells transfected with miR-152-3p-mimcs. (**G**) Luciferase reporter assay detected luciferase activity of wild and mutant LINC00221. (**H,I**). RNA pull down and RIP assay verified the interaction between LINC00221 and miR-152-3p. (**J,K**). Subcellular fraction assay and FISH assay (scale bar: 10 μm) were carried out to detect the subcellular location of LINC00221 in ALL cells. *P<0.05, **P<0.01.

As demonstrated in GEPIA database, LINC00221 was remarkably down-regulated in acute myeloid leukemia tissues (Figure 1A). Then, we sought to explore whether LINC00221 is expressed at a low level in ALL cells. Through qRT-PCR, we observed that LINC00221 was also remarkably down-regulated in ALL cells (Jurkat, CCRF-CEM, CEM/C1, 1301) in contrast with HEK 293T cells (Figure 1B). Since CCRF-CEM and CEM/C1 contained the lowest expression of LINC00221, they were chosen for the following experiments. Then, we explored the potential mechanism of LINC00221 in ALL. Increasing studies revealed that lncRNAs could sequester miRNAs in cancers. Thus, we sought to identify miRNAs which could bind to LINC00221. Based on LncBase database [13], five miRNAs which got the highest binding scores were selected out. Among them, miR-152-3p and miR-1273g-5p were significantly pulled down by biotinylated LINC00221 probe (Figure 1C). Next, we detected the expression of miR-152-3p and miR-1273g-5p in ALL cells and the results from qRT-PCR demonstrated that only miR-152-3p was up-regulated in ALL cells while miR-1273g-5p expression did not show significance in ALL cells (Figure 1D). Then, binding site between LINC00221 and miR-152-3p were identified and the mutation was designed (Figure 1E). We increased expression of miR-152-3p by transfecting miR-152-3p mimics and the transfection efficiency was verified by qRT-PCR analysis (Figure 1F). Then, the luciferase reporter assay disclosed that decreased luciferase activity of wild LINC00221 and unchanged luciferase activity of mutant LINC00221 by miR-152-3p mimics (Figure 1G). Also, RNA pull down assay unveiled that LINC00221 was remarkably pulled down by biotinylated wild miR-152-3p while biotinylated mutant miR-152-3p took no effects on LINC00221 enrichment (Figure 1H). RIP assay further demonstrated that both LINC00221 and miR-152-3p were significantly enriched in Anti-Ago2 group but not Anti-IgG group (Figure 1I). Moreover, cytoplasm/nucleus fraction assay and FISH assay demonstrated that LINC00221 was mainly distributed in cytoplasm of ALL cells (Figure 1J,K), suggesting that LINC00221 exerted its regulatory functions at post-transcriptional level. Based on these findings, we concluded that LINC00221 was down-regulated in ALL cells and bound to miR-152-3p.

### MiR-152-3p restored LINC00221-mediated anti-proliferation and pro-apoptosis effects in ALL cells

**Figure 2:**
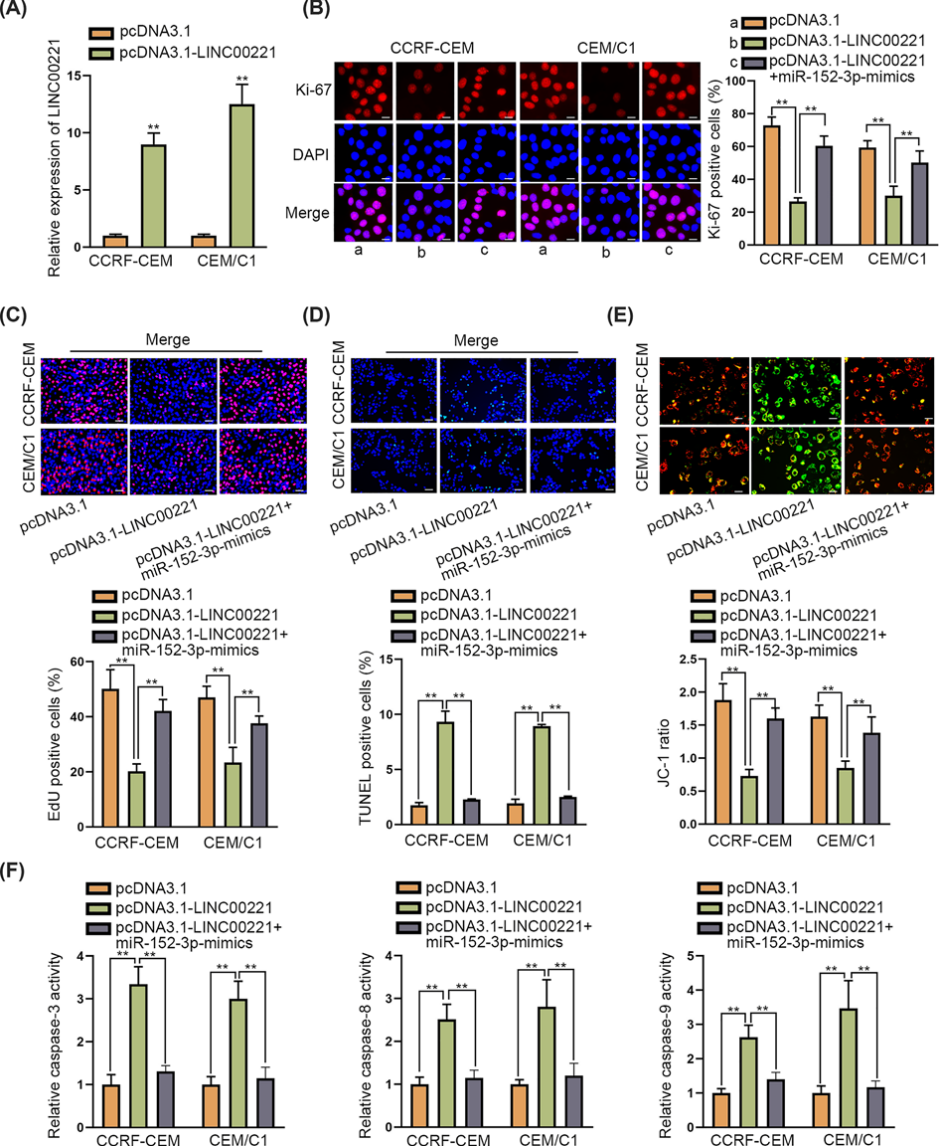
MiR-152-3p restored LINC00221-mediated anti-proliferation and pro-apoptosis effects in ALL cells (**A**) qRT-PCR verified overexpression efficiency of ATP2A2. (**B,C**) Ki-67 immunofluorescence staining assay (scale bar: 20 μm) and EdU assay (scale bar: 100 μm) detected ALL cell proliferation ability in pcDNA3.1-LINC00221 group or in pcDNA3.1-LINC00221+miR-152-3p-mimics group. (**D**–**F**) TUNEL (scale bar: 100 μm), JC-1 (scale bar: 20 μm) and detection of caspase-3/8/9 activity examined ALL cell apoptosis ability in pcDNA3.1-LINC00221 group or in pcDNA3.1-LINC00221+miR-152-3p mimics group. **P<0.01.

To explore the function of LINC00221 in ALL cells, pcDNA3.1-LINC00221 was transfected into ALL cells and the transfection efficiency was verified by qRT-PCR analysis (Figure 2A). Then, Ki-67 immunofluorescence staining assay and EdU assay disclosed that LINC00221 overexpression inhibited ALL cell proliferation but this suppressive effect was restored by co-transfecting miR-152-3p-mimics (Figure 2B,C). Moreover, TUNEL and JC-1 assay revealed that ALL cell apoptosis ability enhanced by LINC00221 overexpression was decreased by co-transfection of miR-152-3p-mimics (Figure 2D,E). Also, miR-152-3p overexpression restored LINC00221-mediated accelerative effect on caspase-3/8/9 activity (Figure 2F). Taken together, LINC00221 exerted anti-proliferation and pro-apoptosis effects on ALL cells while miR-152-3p counteracted theses effects on ALL cells.

### LINC00221 served as a ceRNA to up-regulate ATP2A2

**Figure 3:**
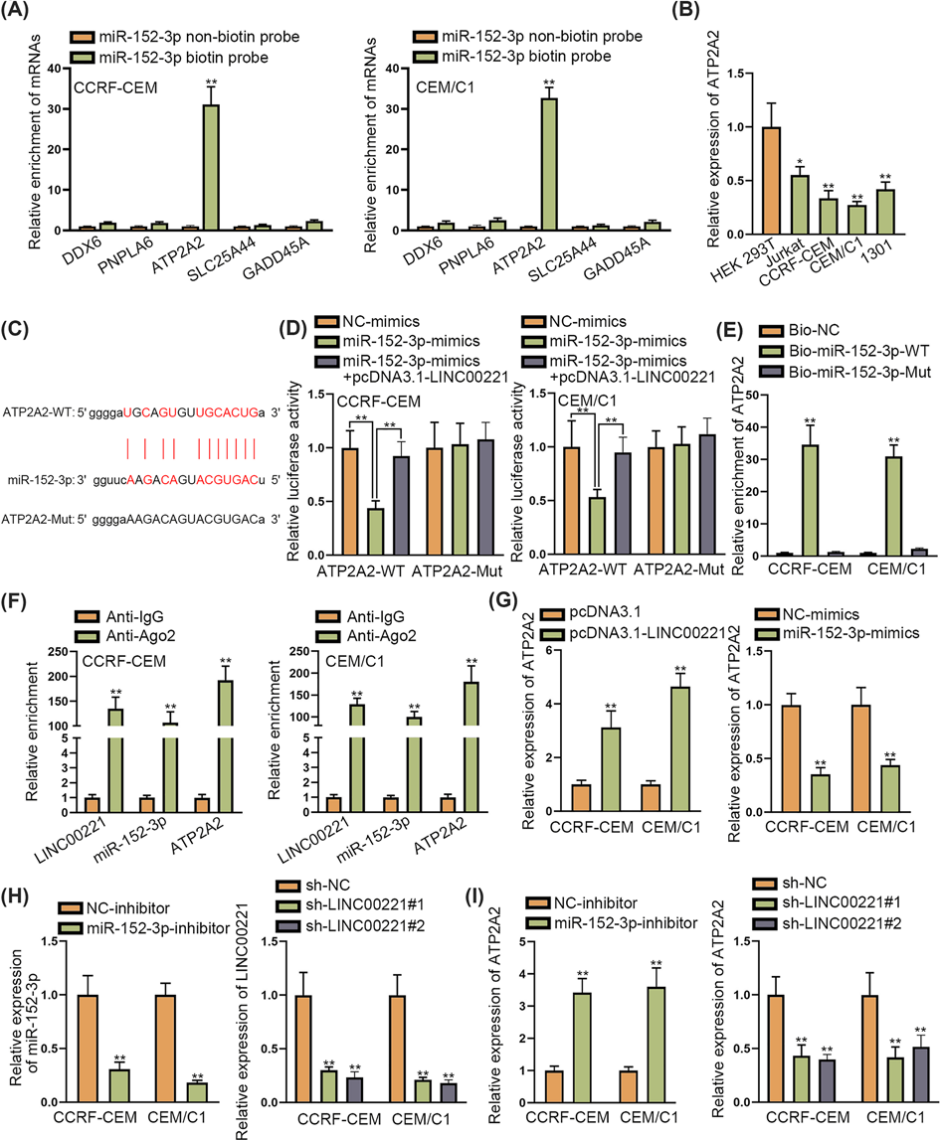
LINC00221 served as a ceRNA to up-regulate ATP2A2 (**A**) RNA pull down assay examined expression of potential target genes pulled down by miR-152-3p. (**B**) qRT-PCR determined ATP2A2 expression in ALL cells and HEK-293T cells. (**C**) Binding sites of wild/mutant ATP2A2 and miR-152-3p. (**D**) Luciferase reporter assay detected luciferase activity of wild and mutant ATP2A2. (**E**) RNA pull down assay detected enrichment of ATP2A2 pulled down by biotin-labeled wild/mutant miR-152-3p. (**F**) RIP assay detected miR-152-3p, ATP2A2 and LINC00221 enrichment in Ago2 and IgG group. (**G**) qRT-PCR examined effects of LINC00221 and miR-152-3p overexpression on ATP2A2 expression in ALL cells. (**H**) qRT-PCR examined knockdown efficiency of miR-152-3p and LINC00221 in ALL cells. (**I**) qRT-PCR examined influence of miR-152-3p and LINC00221 depletion on ATP2A2 expression. *P<0.05, **P<0.01.

To further support ceRNA hypothesis, we explored the target gene of miR-152-3p. The starBase database [14] was used for searching for the potential targets for miR-152-3p. As screened by strict stringency in CLIP data, high stringency in Degradome data and six predicted programs of microT, miRanda, miRmap, PITA, PicTar and TargetScan, five potential targets of miR-152-3p were identified. Among them, only ATP2A2 was significantly pulled down by biotinylated miR-152-3p (Figure 3A). Next, we detected expression of ATP2A2 in ALL cells and it was revealed that ATP2A2 was significantly down-regulated in ALL cells (Figure 3B). The binding site of the ATP2A2 and miR-152-3p are demonstrated in Figure 3C. Luciferase reporter assay revealed that luciferase activity of ATP2A2-WT reporter reduced by miR-152-3p overexpression was recovered by co-transfection of pcDNA3.1-LINC00221 while that of ATP2A2-Mut reporter was not influenced (Figure 3D). Further, ATP2A2 was remarkably pulled down by biotinylated wild miR-152-3p and biotinylated mutant miR-152-3p could hardly pull down ATP2A2 (Figure 3E). RIP assay indicated that ATP2A2, miR-152-3p and LINC00221 were significantly enriched in Anti-Ago2 group but not in Anti-IgG group (Figure 3F). Subsequently, we detected the influence of pcDNA3.1-LINC00221 and miR-152-3p-mimics on ATP2A2 expression. The results revealed that pcDNA3.1-LINC00221 caused increased expression of ATP2A2 while miR-152-3p-mimics caused decreased expression of ATP2A2 (Figure 3G). Moreover, we knocked down the expression of miR-152-3p and LINC00221 in ALL cells and the knockdown efficiency was verified by qRT-PCR analysis (Figure 3H). Depletion of miR-152-3p elevated ATP2A2 expression while LINC00221 depletion decreased ATP2A2 expression (Figure 3I). Based on these findings, we concluded that miR-152-3p targeted ATP2A2 and that LINC00221 sponged ATP2A2 to elevate ATP2A2 expression in ALL cells.

### ATP2A2 was required in LINC00221-mediated effects in ALL cells

**Figure 4:**
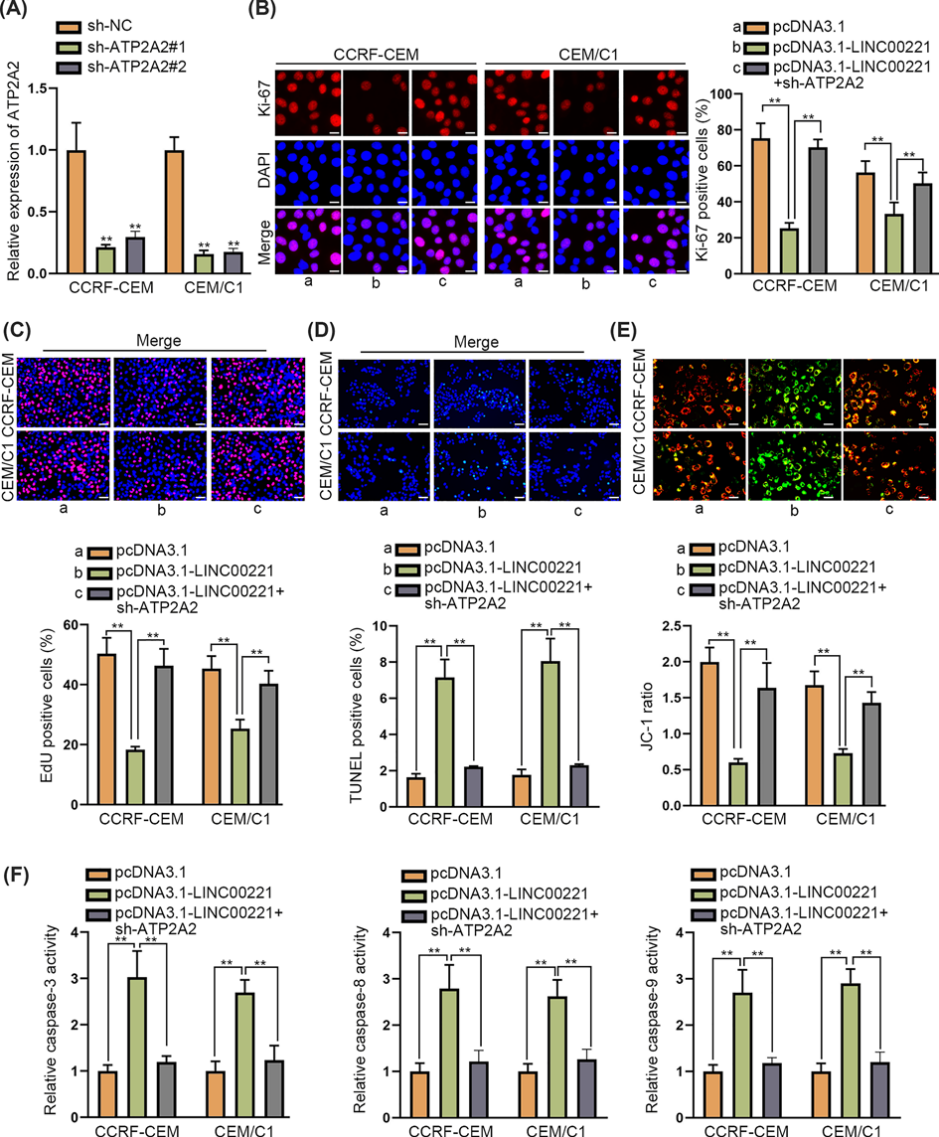
ATP2A2 was required in LINC00221-mediated effects in ALL cells (**A**) qRT-PCR verified knockdown efficiency of ATP2A2. (**B,C**) Ki-67 immunofluorescence staining assay (scale bar: 20 μm) and EdU assay (scale bar: 100 μm) detected cell proliferation ability of ALL cells transfected with pcDNA3.1-LINC00221 or co-transfected with pcDNA3.1-LINC00221+ sh-ATP2A2. (**D**–**F**) TUNEL (scale bar: 100 μm), JC-1 (scale bar: 20 μm) and detection of caspase-3/8/9 activities were performed to examine cell apoptosis ability in ALL cell transfected with pcDNA3.1-LINC00221 or co-transfected with pcDNA3.1- LINC00221+sh-ATP2A2. **P<0.01.

To further verify whether ATP2A2 was involved in LINC00221-mediated anti-proliferation and pro-apoptosis effects on ALL cells, the rescue assays were conducted. At first, knockdown efficiency of ATP2A2 was verified by qRT-PCR analysis (Figure 4A). Then, it was disclosed in Ki-67 immunofluorescence staining assay and EdU assay that ATP2A2 depletion countervailed LINC00221-caused suppressive effect on ALL cell proliferation (Figure 4B,C). Also, LINC00221-mediated promoting effect on ALL cell apoptosis was restored by ATP2A2 depletion in TUNEL assay, JC-1 assay and caspase-3/8/9 activity assay (Figure 4D–F). According to these findings, we concluded that ATP2A2 depletion abrogated LINC00221-mediated anti-proliferation and pro-apoptosis effects on ALL cells.

## Discussion

ALL is a heterogeneous disease with high incidence rate in children. Accumulating studies have uncovered function of molecules in ALL. MiR-31 is a putative biomarker for screening children B-cell ALL [15]. ARIEL activates the expression of ARID5B and positively modulates TAL1-activated transcriptional program in T-cell ALL [16]. The present study started from LINC00221, which was verified to be down-regulated in AML tissues based on GEPIA database. We figured out that LINC00221 was obviously down-regulated in ALL cells in contrast with control cells. LncRNAs were reported to bind with miRNAs in various diseases, including leukemia. LncRNA GAS5 facilitates tumorigenesis and metastasis of B lymphocytic leukemia by acting as a sponge of miR-222 [17]. LncRNA SNHG3 interacted with miR-758-3p and facilitated cell growth in acute myeloid leukemia [18]. LncRNA KCNQ1OT1 sponges miR-326 to control cell proliferation and differentiation in acute myeloid leukemia [19]. We then identified miR-152-3p as the miRNA which bound to LINC00221 by a series of mechanism assays including luciferase reporter assay, RIP assay and RNA pull down assay. MiR-152-3p was obviously up-regulated in ALL cells. Next, the function assays uncovered that LINC00221 inhibited ALL cell proliferation and boosted ALL cell apoptosis while miR-152-3p reversed LINC00221 mediated effects on ALL cells. The oncogenic role of miR-152-3p has been revealed. MiR-152-3p facilitates chronic myeloid leukemia development via p27 inhibition [20]. Melatonin targets miR-152-3p and represses angiogenic factors in triple-negative breast cancer [21]. Intriguingly, MiR-152-3p was also a tumor inhibitor in glioma [22], prostate cancer [23] and triple-negative breast cancer [21].

Moreover, we figured out that miR-152-3p targeted ATP2A2 in ALL cells. MiR-152-3p negatively regulated ATP2A2 expression and LINC00221 positively regulated ATP2A2 expression via sponging miR-152-3p. Subsequently, we verified that LINC00221 posed anti-proliferation and pro-apoptosis effects on ALL cells via down-regulating ATP2A2. High expression of ATP2A2 was identified to be positively correlated with favorable prognosis of astrocytoma [24].

The ceRNA pattern in leukemia was previously uncovered. As is revealed by Pouyanrad et al. [25], miR-335-3p is sponged by NEAT1 and MALAT1 and is associated with poor prognosis in ALL through targeting ABCA3. MEG3 served as an anti-oncogene in T-cell lymphoblastic lymphoma through MEG3/miR-214/AIFM2 pathway [26]. LncRNA SNHG16 sponges miR-497-5p and elevates PIM1 expression, boosting cell proliferation and suppressing apoptosis in diffuse large B-cell lymphoma cells [27]. Up-regulation of LINC00641 contributes to cell growth and migration in acute myeloid leukemia through modulation on miR-378a/ZBTB20 axis [28].

On the whole, present study uncovered a novel ceRNA network in ALL cells. LINC00641 contributed to ALL cell apoptosis and inhibited ALL cell proliferation via sponging miR-152-3p and up-regulating ATP2A2, shedding a new insight into ALL treatment.

## Abbreviations

Ago2: argonaute 2
ALL: acute lymphoblastic leukemia
AML: acute megakaryoblastic leukemia
ATP2A2: ATPase sarcoplasmic/endoplasmic reticulum Ca^2+^ transporting 2
ceRNA: competing endogenouse RNA
DAPI: 4’,6-diamidino-2-phenylindole
EDU: 5-Ethynyl-2’-Deoxy-uridine
Fish: Fluorescence in situ hybridization
GAPDH: glyceraldehyde-3-phosphate dehydrogenase
LINC00221: long intergenic non-protein coding RNA 221
lncRNA: long non-coding RNA
NC: negative control
qRT-PCR: quantitative real-time PCR
RIP: RNA immunoprecipitation
